# Rationale for concurrent chemoradiotherapy for patients with stage III non-small-cell lung cancer

**DOI:** 10.1038/s41416-020-01070-6

**Published:** 2020-12-08

**Authors:** John Conibear, on behalf of AstraZeneca UK Limited

**Affiliations:** Department of Clinical Oncology, St. Bartholomew’s Hospital, London, UK

## Abstract

When treating patients with unresectable stage III non-small-cell lung cancer (NSCLC), those with a good performance status and disease measured within a radical treatment volume should be considered for definitive concurrent chemoradiotherapy (cCRT). This guidance is based on key scientific rationale from two large Phase 3 randomised studies and meta-analyses demonstrating the superiority of cCRT over sequential (sCRT). However, the efficacy of cCRT comes at the cost of increased acute toxicity versus sequential treatment. Currently, there are several documented approaches that are addressing this drawback, which this paper outlines. At the point of diagnosis, a multidisciplinary team (MDT) approach can enable accurate assessment of patients, to determine the optimal treatment strategy to minimise risks. In addition, reviewing the Advisory Committee on Radiation Oncology Practice (ACROP) guidelines can provide clinical oncologists with additional recommendations for outlining target volume and organ-at-risk delineation for standard clinical scenarios in definitive cCRT (and adjuvant radiotherapy). Furthermore, modern advances in radiotherapy treatment planning software and treatment delivery mean that radiation oncologists can safely treat substantially larger lung tumours with higher radiotherapy doses, with greater accuracy, whilst minimising the radiotherapy dose to the surrounding healthy tissues. The combination of these advances in cCRT may assist in creating comprehensive strategies to allow patients to receive potentially curative benefits from treatments such as immunotherapy, as well as minimising treatment-related risks.

## Background

When treating patients with locally advanced, stage III, non-small-cell lung cancer (NSCLC), those with a good performance status (PS, defined as an Eastern Cooperative Oncology Group [ECOG] PS 0–1) and disease encompassable within a radical treatment volume should be considered for definitive concurrent chemoradiotherapy (cCRT).^1–6^ This guidance is based on the results of several preclinical studies documenting beneficial interactions between radiation and chemotherapy, as well as two large Phase 3 randomised studies and two meta-analyses (in patients who predominantly had stage III NSCLC), which demonstrated the superiority of cCRT over sequential chemoradiotherapy (sCRT, chemotherapy followed by full-dose radiotherapy, with sequential defined as chemotherapy followed by radiotherapy).^7–12^ Currently in the United Kingdom (UK), 45% of stage III NSCLC patients with curative radiation doses (*n* = 716) are treated with cCRT, compared with 55% (*n* = 391) who receive sCRT.^13^ For comparison, an observational study of national registries demonstrated that in Belgium and The Netherlands, 35 and 55% of patients received cCRT, respectively.^14^

Several preclinical studies have outlined the synergistic benefits between radiation and chemotherapy drugs, such as cisplatin, carboplatin and cisplatin plus etoposide.^12^ These early studies outlined the radiosensitising properties of chemotherapy drugs, such as cisplatin, as well as how the close temporal administration of the two can enhance antitumour efficacy, but with high toxicity costs. Although several mechanisms of action have been proposed following in vitro and in vivo studies, platinum-radiation interactions are complex and not fully comprehended at this time. One possibility proposed is reduced recovery from radiation-induced, potentially lethal or sublethal damage, when cisplatin is present.^12^ In addition, cells can arrest in the second growth phase following radiotherapy, and are shown to be hypersensitive to the cytotoxic effect of etoposide.^11^ Early clinical studies of CRT in NSCLC examined whether a sequential approach to treatment delivery was useful to maximise both locoregional and micrometastatic disease control, whilst minimising the risks of cumulative toxicity. Despite modest improvements with sCRT, two large Phase 3 randomised studies demonstrated superiority of cCRT over sCRT.^8,9^

## Review of key studies comparing concurrent and sequential chemoradiotherapy

In the Radiation Therapy Oncology Group trial (RTOG 9410), 610 patients with unresectable stage III NSCLC were randomised to one of three arms: two cycles of cisplatin plus vinblastine with either concurrent or sequential radiotherapy (60 Gy in 30 fractions) or two cycles of cisplatin plus oral etoposide with concurrent radiotherapy delivered twice daily (69.6 Gy in 20 fractions delivered at 1.2 Gy per fraction). Patients who received cCRT with cisplatin plus vinblastine demonstrated improved overall survival (OS) compared with those who received sequential treatment (median OS, 17.0 vs. 14.6 months; HR 0.81; 95% CI 0.66–0.996) at the cost of increased rates of acute grade ≥3 non-haematologic toxicity. Late toxicity rates were similar overall for all arms of the study.^8^

In a second Phase 3 randomised study, 320 patients were randomly assigned to cisplatin, mitomycin and vindesine with a concurrent, split course of thoracic radiotherapy (2 Gy/fraction given 14 times for 3 weeks and then followed by a rest period of 10 days) or to the same chemotherapy regimen followed by a single course of radiotherapy (56 Gy in 28 fractions of 2 Gy each). In both arms of the study, the radiotherapy planning techniques, dose constraints and treatment delivery were the same (either delivered using a linear accelerator [≥4 MeV] or a cobalt-60 machine). The cCRT arm was associated with an improved response rate (84% vs. 66%), median OS (16.5 vs. 13.3 months) and 2- and 5-year survival rates (34.6% vs. 27.4% and 15.8% vs. 8.9%, respectively) compared with the sCRT arm. Treatment-related toxicity included myelosuppression, which occurred more frequently in patients in the concurrent arm compared to the sequential arm (*p* = 0.0001). There was no significant difference in the incidence of other toxicities, including oesophagitis, between the two treatment arms.^9^

A meta-analysis published in 2010 demonstrated the superiority of cCRT over radiotherapy alone or sCRT.^7^ The meta-analysis included 19 randomised studies (with over 2,700 patients) and reported a significantly reduced overall risk of death (HR 0.71; 95% CI 0.64–0.80) and improved progression-free survival (PFS) at any site (HR 0.69; 95% CI 0.58–0.81) for those receiving cCRT compared with radiotherapy alone.^7,15^ These improvements were at the cost of increased toxicity with higher rates of acute oesophagitis, neutropenia and anaemia in patients receiving cCRT over sCRT.^7^ The meta-analysis also analysed six trials (1024 patients) comparing cCRT versus sCRT. A significant benefit was shown in OS (HR 0.74; 95% CI 0.62–0.89) for cCRT. This survival improvement equated to a 10% absolute survival benefit at 2 years for cCRT.^7^ Again, this was at the cost of toxicity with increased rates of severe oesophagitis (relative risk [RR] 4.96; 95% CI 2.17–11.37) and a non-significant increase in treatment-related deaths (4% vs. 2%) reported in the cCRT arm versus the sCRT arm (RR 2.02; 95% CI 0.90–4.52), respectively.^7^

A subsequent meta-analysis (1205 patients) by Auperin et al. analysed randomised trials directly comparing cCRT versus sCRT and demonstrated the superiority of cCRT, which was shown to improve OS in patients with locally advanced NSCLC (HR 0.84; 95% CI 0.74–0.95; *p* = 0.004).^10^ This improvement in OS was at the cost of increased acute toxicity, particularly oesophagitis; cCRT increased acute oesophagitis (grade 3–4) from 4 to 18% with a RR of 4.9 (95% CI 3.1–7.8; *p* < 0.001).^10^ Two further randomised Phase 3 studies that compared sCRT versus cCRT in patients with unresectable NSCLC failed to show a statistically significant difference in OS between arms.^16,17^ The first of these studies was not sufficiently powered and was closed early; however, radical radiotherapy (66 Gy), given concurrently with daily low-dose cisplatin, or after two courses of gemcitabine plus cisplatin, was well tolerated with 2-year OS rates of 34 and 39%, and 3-year OS rates of 22 and 34%, respectively, for sCRT versus cCRT.^16^ In the cCRT arm, oesophagitis occurred in nine patients (14%) at grade 3 and in two patients (3%) at grade 4, while in the sCRT arm, it occurred in four patients (5%) at grade 3 and no patients at grade 4. Acute haematological toxicity was more common in the sCRT arm compared with the cCRT arm.^16^ The second randomised study failed to show a statistically significant improvement in OS; however, it did reveal clinically important differences in the median 2-, 3- and 4-year OS rates with a trend in favour of cCRT, suggesting that this is the optimal strategy for patients with locally advanced NSCLC.^17^ The culmination of these studies led to cCRT to be considered the standard of care for patients with unresectable stage III NSCLC.

## Determining treatable areas for radiotherapy

Determining the optimal treatment plan for a patient with NSCLC requires an accurate assessment of their overall fitness, medical comorbidities, cardiorespiratory reserve, genomic background, tumour stage and mutation status.^18^ Once this assessment is completed, it is then possible to develop the optimal treatment strategy for the patient. When selecting stage III NSCLC patients for CRT, it is important to consider that certain aspects of therapy remain controversial. Hence, a multidisciplinary team (MDT) approach that includes expert opinions from thoracic surgeons, clinical and medical oncologists, radiologists, nuclear medicine physicians and pathologists^18^ is necessary to ensure that all patients are offered optimal treatments based on their surgical operability, performance status, stage and extent of disease.

When pathological mediastinal lymph node (N2) disease is evident at the time of diagnosis, a combined-modality approach is normally recommended if the patient is deemed radically treatable. Due to the presence of nodal disease, such patients are considered high risk for both local and distant recurrence, and surgical resection as the sole treatment modality is considered inadequate. Consequently, the most common approach to managing patients with confirmed N2 nodal involvement is cCRT, using a platinum doublet-based chemotherapy combined with a radical dose of radiotherapy. Within this group of N2 patients, there is a highly selected subset of patients who may be considered for surgery following preoperative chemotherapy and/or CRT, with existing guidelines suggesting that such patients should have minimal N2 disease.^2^ It is currently debated whether surgery should be considered for the subset of N2 patients who require pneumonectomy (those with extensive mediastinal N2 infiltration), due to the high mortality rates associated with the procedure.^19^ For patients with N3 or T4 surgically unresectable tumours, the standard approach to management is cCRT.^3^ Hence, radiotherapy in the context of cCRT plays a major role in the radical treatment of NSCLC patients with locally advanced disease. Guidelines have been developed by the ACROP committee, on behalf of the European Society for Radiotherapy and Oncology (ESTRO) for the treatment of locally advanced NSCLC.^20^ These guidelines provide recommendations for target volume (TV) and organ-at-risk (OAR) delineation for standard clinical scenarios with definitive CRT (and adjuvant radiotherapy) for locally advanced NSCLC, and also give a comprehensive guide on how to plan a patient’s radical radiotherapy from pre-treatment imaging to planning computed tomography (CT) acquisition and optimal gross tumour volume (GTV), clinical target volume (CTV), planning target volume (PTV) and OAR definitions.^20^

## Treatment statistics and toxicity profiles for concurrent versus sequential chemoradiotherapy

As discussed, cCRT leads to improvements in efficacy, at the cost of increased acute toxicity, compared with sCRT.^7–10,15^ Following the publication of the meta-analyses described above, Koning et al. conducted a systematic literature review to evaluate the toxicity of cCRT for locally advanced NSCLC;^21^ 13 studies in patients with stage III NSCLC were identified, which featured mono- or polychemotherapy schedules as single dose, double dose, triple high-dose or daily cisplatin-containing chemotherapy.^21^ From these studies, acute (grade ≥ 3) oesophagitis was observed in up to 18% of the patients, and high-dose cisplatin regimens resulted in more frequent haematologic toxicity, nausea and vomiting (at least four trials with ≥16%) compared with other chemotherapy regimens.^21^ The review concluded that the toxicity profile was more favourable with low-dose chemotherapy schedules, and cCRT with daily cisplatin monochemotherapy resulted in more favourable acute and late toxicity compared with cCRT with single high-dose chemotherapy, doublets or triplets.^21^

A further retrospective study from The Netherlands of 154 patients with inoperable stage III NSCLC receiving cCRT also demonstrated good efficacy when low-dose chemotherapy was delivered concurrently with conformal, intensity-modulated radiation therapy (IMRT), or volumetric-modulated arc therapy (VMAT). Patients who received 66 Gy (24 fractions of 2.75 Gy) with low-dose daily cisplatin (6 mg/m^2^) had a 5-year survival rate of 40%.^22^ In addition, Arrieta et al. also reported that the use of induction gemcitabine plus carboplatin followed by cCRT utilising gemcitabine led to unacceptably high rates of pulmonary toxicity (39.1% of patients had grade 3–5 toxicity), despite improved response rates.^23^ Based on all these findings, platinum doublet chemotherapy remains the standard of care when delivering cCRT, but there is no clear evidence to support one regimen over another.

The UK Phase 2 SOCCAR trial, which compared sequential versus concurrent chemotherapy and radical hypofractionated radiotherapy in 130 patients with unresectable stage III NSCLC and good performance status (ECOG PS 0–1), also confirmed that it was feasible to deliver accelerated hypofractionated radiotherapy with chemotherapy to these patients. Patients recruited to the trial received concurrent cisplatin and vinorelbine chemotherapy with a minimum standard of conformally planned radiotherapy (4D-CT radiotherapy planning was used by one participating centre, otherwise IMRT was not routinely used). The incidence of at least one serious adverse event (AE) was similar in both arms. Rates of grade 3–5 AEs were 32% in the cCRT arm and 41% in the sCRT arm, with oesophagitis reported in 8.8% and 8.5% of patients and pneumonitis in 3.1% and 5.2% of patients, respectively.^24^ The conclusion of the SOCCAR trial was that the encouraging 2-year survival rates (50% in the concurrent arm) suggest that a 4-week hypofractionated regimen of radiotherapy should be compared with conventionally fractionated radiotherapy in an adequately powered randomised controlled Phase 2 trial.^24^

## Comparison of United Kingdom lung cancer guidelines with European Union standards

Within the UK, there is no shortage of guidance on the optimal management of stage III NSCLC patients as both national (e.g. National Institute for Health and Care Excellence [NICE] and The Royal College of Radiologists and British Thoracic Society) and regional (e.g. London Cancer Alliance) guidelines exist.^1,4,6,18,25^ All provide clear and consistent guidance on the management of patients with locally advanced NSCLC, including the use of cCRT. A summary of UK national guidance is provided in Table 1, with comparison to the European Society for Medical Oncology (ESMO) locally advanced NSCLC guidelines.^2,6^ These comparisons reveal strong correlation on the optimal management of locally advanced NSCLC patients, and it is reassuring that cCRT is also considered the treatment of choice in patients evaluated as having unresectable stage IIIA and IIIB disease based on published ESMO consensus guidance.^2,3^ Comparison of ESMO guidance,^2,3^ with that from NICE,^5^ The Royal College of Radiologists^25^ and the British Thoracic Oncology Group^4^ reveals no significant variations in standards, and therefore highlights the consensus agreement that exists on the optimal management of these patients across the European Union (EU).

**Table 1:** Summary of the UK national guidelines for optimal management of stage III NSCLC patients.

Topic	RCR guidance statement^25^	Evidence level^25^	NICE guidance statement^6^	Supporting references	Consensus with ESMO guidelines^2^
cCRT versus sCRT or radiotherapy alone	cCRT has been demonstrated in meta-analyses to give superior outcomes when compared with sCRT or radiotherapy alone	1a^a^/A^b^	There were limited data available on whether continuous radiotherapy with concurrent chemotherapy was more effective than alternating radiotherapy and chemotherapy	^4,7,10,15,24,25^	cCRT generally gives significantly better OS results than sCRT and radiotherapy protocols in unresectable IIIA and IIIB disease
CRT versus surgery	No recommendations provided for stage III	-	Consider CRT for patients with stage II or III NSCLC who are not suitable or decline surgery. Balance potential benefit in survival with the risk of additional toxicities. For people with operable stage IIIA–N2 NSCLC who can have surgery and are well enough for multimodality therapy, consider chemoradiotherapy with surgery	^4,5^	cCRT is the treatment of choice in patients evaluated as unresectable in stage IIIA and IIIB
Elderly patients	Elderly patients with good performance status (0–1) and few comorbidities derive equal benefit from concurrent therapies as their younger counterparts	1b^a^	No recommendations provided	^25,80–83^	Age itself has not been shown to influence outcome following definitive cCRT. However, data are limited for the elderly population and, in particular, in patients above 75 years of age
Neoadjuvant or adjuvant chemotherapy	There is no evidence of benefit for chemotherapy delivered either neoadjuvantly or adjuvantly to those receiving cCRT	1b^a^	No recommendations provided for stage III	^25^	In the stage III disease CRT strategy, there is no evidence for further induction or consolidation chemotherapy
Dose fractionation of concurrent radiotherapy	55 Gy in 20 fractions over 4 weeks with cisplatin and vinorelbine, 60 Gy in 30 fractions over 6 weeks with cisplatin and etoposide and 66 Gy in 33 fractions over 6.5 weeks with cisplatin and etoposide	A^b^	If conventionally fractionated radical radiotherapy is used, offer either 55 Gy in 20 fractions over 4 weeks or 60–66 Gy in 30–33 fractions over 6–6½ weeks. Accelerated radiotherapy fractionation schedules seem to improve outcomes in NSCLC	^24–26,28^	Promising outcome is achieved with accelerated radiotherapy. A potential radiation schedule could be the delivery of 66 Gy in 24 fractions
cCRT toxicity	Concurrent schedules have a higher incidence of grade ≥3 oesophageal toxicities	1b^a^	No recommendations provided	^7,10,15–17,25^	No recommendations provided

*(c)(s)CRT* (concurrent)(sequential) chemoradiotherapy, *Gy* grey, *NSCLC* non-small-cell lung cancer, *OS* overall survival.

^a^The Oxford Centre for evidence-based medicine levels of evidence.^84^

^b^Guidelines on the radical management of patients with lung cancer.^4^

This table was created by the author, using guidance from refs. ^4,5,7,10,15,17,24–26,28,80–84^.

(c)(s)CRT, (concurrent)(sequential) chemoradiotherapy; Gy, grey; NSCLC, non-small-cell lung cancer; OS, overall survival.

## Optimal chemotherapy regimens and radiotherapy dose fractionation for concurrent chemoradiotherapy

The choice of chemotherapy can additionally be important to the outcome of cCRT. According to the ESMO guidelines “in the absence of contraindications, the optimal chemotherapy to be combined with radiation in stage III NSCLC should be based on cisplatin”.^2^ Current evidence is inconclusive on use of cisplatin as a single agent, but it may be combined with etoposide, vinorelbine or other vinca alkaloids, based on most comparative clinical trials of cCRT and sCRT when treatment has curative intent.^2,10^ If a patient has specific comorbidities, other treatment options that can be considered include carboplatin-based cCRT. In terms of regimen delivery, ESMO guidelines recommend that “in the stage III disease chemoradiotherapy strategy, two to four cycles of concomitant chemotherapy should be delivered”.^2^

In terms of radiotherapy, multiple studies have examined the use of alternative dose-fractionation schedules to improve outcomes for NSCLC patients. Approaches have included hyperfractionation (two or three fractions per day with a lower dose per fraction than the standard 2 Gy), accelerated fractionation (the overall duration of treatment is shortened, but the fraction size and total dose is kept the same), hypofractionation (where a larger daily fraction size is used than the standard 2 Gy), dose escalation (where the total dose is increased) or combinations of these different approaches.^24,26–33^

One of the earliest studies to assess the effect of total radiation dose on outcomes in patients with NSCLC was the RTOG 7301 study that examined the effects of dose escalation on tumour control by randomising 365 stage III NSCLC patients to one of four treatment arms, with local tumour response rates being significantly improved with the highest dose (60 Gy in 30 daily fractions, over 5 days a week for 6 weeks; 56% response rate), although survival was similar between arms.^33^ This study was the first to establish 60 Gy in 30 daily fractions, over 5 days a week for 6 weeks, as the standard radiotherapy dose-fractionation schedule for NSCLC.^30,33^

The Phase 3 RTOG 0617 trial examined whether high-dose (74 Gy in 37 daily fractions) radiotherapy offers an advantage over standard-dose (60 Gy in 30 daily fractions) radiotherapy, whether the method of treatment delivery (IMRT vs. 3D-conformal radiotherapy) improves patient outcomes and whether the addition of cetuximab to carboplatin and paclitaxel has any beneficial effects.^30^ Between November 2007 and November 2011, 544 stage III NSCLC patients from the United States and Canada were enrolled in the open-label 2×2 factorial trial and were randomised 1:1:1:1 to one of two systemic regimens (weekly carboplatin [AUC 2] plus paclitaxel [45 mg/m^2^] with or without cetuximab) and to either standard-dose (60 Gy) or high-dose radiotherapy (74 Gy), delivered using either 3D-conformal radiotherapy techniques or by IMRT. Two weeks after their chemoradiation finished, all enrolled patients received two 21-day cycles of consolidation chemotherapy with full doses of carboplatin (AUC 6) and paclitaxel (200 mg/m^2^).^30^ Unexpectedly, the high-dose (74-Gy) arm was associated with significantly shorter OS and an increased risk of death compared with the standard-dose radiotherapy arm (median OS, 20 vs. 29 months; HR 1.38; 95% CI 1.09–1.76).^30^ Both the radiation and cetuximab comparisons crossed prespecified futility boundaries. The reasons for why the RTOG 0617 trial failed to show a benefit for radiotherapy dose escalation seem to be multifactorial. The escalated radiotherapy dose in the experimental arm (74 Gy) resulted in less patients completing their planned treatment compared with the control arm (64% vs. 70%, respectively), higher rates of treatment planning non-compliance in the 74-Gy arm (26% vs. 17%) and higher doses of radiotherapy to the heart in the 74-Gy arm.^30^ Bradley et al. also noted that fewer patients in the high-dose arm completed consolidation chemotherapy and hypothesised that 74 Gy given over 7.5 weeks allowed increased tumour repopulation to occur.

Despite these shortcomings, the RTOG 0617 trial was the first Phase 3 trial to permit IMRT in NSCLC and demonstrated that IMRT improved outcomes compared with 3D-conformal radiotherapy. IMRT showed similar survival and locoregional control rates to 3D-conformal radiotherapy, but lower rates of grade ≥ 3 pneumonitis and lower radiation doses to the heart.^30,34^ Movsas et al. also reported improved quality of life in patients on RTOG 0617 at 3- and 12 months following IMRT compared with 3D-conformal radiotherapy planning at the plenary session of the 2013 American Society for Radiation Oncology (ASTRO) Annual Meeting.^35^ The 60-Gy standard therapy arm in the RTOG 0617 trial also achieved a 28.7-month median survival that is a positive improvement when compared with previously reported stage III NSCLC studies; however, it should be noted that 90% of enrolled participants had undergone positron emission tomography (PET) staging prior to treatment, which may have contributed towards this finding. Overall, although the RTOG 0617 study failed to show a benefit from dose escalation, it has highlighted the important impact cardiac radiotherapy doses can have on patient outcomes and the subsequent risk of death.^30^

Despite the results of the RTOG 0617 trial, the issue of radiation dose escalation continues to be controversial due to the study factorial design, such as how patients were selected and the inclusion of regimens with mixed efficacies and toxicities, such as carboplatin, paclitaxel and cetuximab. Important ongoing studies, such as RTOG 1106/ACRIN 6697, a randomised Phase 2 study comparing standard 60-Gy radiation therapy with 80-Gy high-dose radiation therapy using adaptive radiation therapy techniques, and ADSCaN, a randomised Phase 2 study of accelerated, dose-escalated, sequential chemoradiotherapy in stage III NSCLC, could help determine the feasibility, treatment toxicity and survival associated with alternative radiotherapy dosing.^36,37^

## Advances in radiotherapy techniques for NSCLC

For NSCLC, new radiotherapy techniques have evolved allowing higher radiation doses in tumour- positive areas while avoiding high doses in surrounding tissues. The replacement of conventional treatment simulation with CT simulation has been associated with a survival advantage,^38^ as has cone beam CT (CBCT) for image guidance.^39^ Stereotactic ablative radiotherapy (SABR) utilises small margins for positional uncertainty, facilitated by 4D-CT, multiple conformal or intensity- modulated beams or arcs and volumetric image guidance.^40^ IMRT has been created as a highly conformal form of radiotherapy, owing to modern advances in radiotherapy treatment planning software (TPS) and treatment delivery. The integration of onboard CT scanner technology into radiotherapy treatment machines has also enabled clinicians to target tumours more accurately, and has led to the creation of image-guided radiotherapy (IGRT).^41^ By utilising both IMRT and IGRT, it is now possible to treat substantially larger lung tumours with higher radiotherapy doses safely, with greater accuracy, and whilst minimising the radiotherapy dose to the surrounding normal tissues.^41–43^ These evolving technologies could be combined with targeted agents to further enhance systemic therapy regimens, reducing the risk of distant metastases. Incorporating these potential advances with recent developments in disease staging, diagnostic imaging and molecular profiling could create comprehensive investigational strategies to improve outcomes in future stage III NSCLC clinical trials.^44–49^

## Adjuvant immunotherapy after concurrent chemoradiotherapy in stage III NSCLC

The synergistic effect between radiation and immune-checkpoint modulations has been demonstrated in multiple preclinical studies,^50–57^ and more recently in the clinical setting following the publication of the Phase 3 PACIFIC trial results.^49^ The use of immune-checkpoint antagonists, specifically anti-PD-1 and anti-PD-L1 therapeutics, has resulted in improved OS in patients with metastatic lung cancer, and has transformed the therapeutic landscape in the first-^58^ and second-line treatment settings.^59^ More recently, the anti-PD-L1 agent durvalumab (Imfinzi^®^▼; AstraZeneca UK Limited) has also been shown to benefit patients with stage III NSCLC, whose tumours express PD-L1 in ≥ 1% of tumour cells, when administered following cCRT.^49^ The use of immunotherapy after platinum-based CRT seems to offer a therapeutic synergism, which up until now has only been hypothesised.

A rationale for combining immunotherapy with radiation was outlined in a recent editorial by Yip and colleagues.^60^ They cited Gajewski et al.^61^ who recognised that patients with non-immunogenic tumours are unlikely to respond to immunotherapy alone due to both factors intrinsic to the tumour itself, such as its mutational burden,^62^ neoantigen heterogeneity^63^ and tumour microenvironment,^64^ and those related to the patient, including human leukocyte antigen (HLA) type, germ-line polymorphisms in immune cell receptors and gut microbiota impact on the immunogenicity of the tumour.^64^ Yip and colleagues acknowledged that by using such knowledge, Smyth and colleagues could provide a framework to discuss how to best tailor combination therapies to the tumour microenvironment by stratifying them into four types based on the presence or absence of tumour- infiltrating lymphocytes (TILs) and their PD-L1 expression status,^65^ as shown in Table 2. Strategies to promote immunogenic death, which in turn activate the innate immune system to prime T cells, may help to convert the immunogenically “cold” tumours found in Type 2 and 4 microenvironments (Table 2) into tumours with a more “inflamed phenotype”, thereby improving their response to checkpoint modulation.^61^ This may be achieved by combining immune-checkpoint modulators with an oncolytic virus,^66^ chemotherapy (KEYNOTE-021, IMpower131 studies)^67,68^ or radiotherapy.^69^

**Table 2:** Stratification of the tumour microenvironment, based on the presence or absence of tumour-infiltrating lymphocytes (TIL) and PD-L1 expression.

	TILs present	TILs absent
PD-L1 positive	Type 1—adaptive immune resistance present	Type 3—intrinsic induction
PD-L1 negative	Type 4—other suppressor pathways in promoting immune tolerance	Type 2—immune ignorance

This table was created by the author, using guidance from ref. ^65^

In terms of combining immune-checkpoint inhibitors with radiotherapy, there have been several mechanisms proposed regarding the interaction between radiation and the tumour microenvironment. The first mechanism, already known to play an important role in inducing tumour immunogenicity,^70^ is through the release of tumour antigens and molecules collectively known as the “damage associated molecular pattern” (DAMPs),^71,72^ which can activate CD8^+^ cytotoxic T cells via the major histocompatibility complex (MHC) class I loading pathway. A second pathway that could be activated is the “STING” pathway, which upregulates the expression of type 1 interferon,^73^ MHC class 1 molecules and the generation of novel peptides.^74,75^ Other possible mechanisms of radiation-induced tumour microenvironment stimulation that could improve T-cell recruitment may include the generation of appropriate chemokines and increased blood flow.^76–79^

## Conclusions

At the point of diagnosis, a multidisciplinary team (MDT) approach can enable accurate assessment of patients with unresectable stage III NSCLC, to determine the optimal treatment strategy to minimise risks and toxicity. In addition, reviewing the Advisory Committee on Radiation Oncology Practice (ACROP) guidelines can provide clinical oncologists with additional recommendations for outlining target volume and organ-at-risk delineation for standard clinical scenarios in definitive cCRT (and adjuvant radiotherapy). Modern advances in radiotherapy treatment planning software and treatment delivery mean that radiation oncologists can now treat substantially larger unresectable tumour and nodal volumes with higher radiotherapy doses and greater accuracy, whilst minimising unwanted radiotherapy doses to the surrounding normal healthy tissues. Furthermore, the combination of advances in both cCRT and immune drug therapy has led to an era of emerging treatments for unresectable stage III NSCLC. We can now routinely offer a more efficacious, evidence-based, radical intent treatment strategy to our stage III NSCLC patients.

## Data Availability

Not applicable.

## Appendix

**Prescribing information**

IMFINZI^®^ ▼(durvalumab) 50 mg/ml solution for infusion.

https://medicines.astrazeneca.co.uk/content/dam/multibrand/uk/en/prescribinginformation/imfinzi-pi.pdf.
